# “My Baby’s Sleep”: A Quality Improvement Project to Promote Safe and Healthy Sleep Practices

**DOI:** 10.7759/cureus.75672

**Published:** 2024-12-13

**Authors:** Ana Teresa Guerra, Sara Completo, Andreia F Ribeiro, Daniela David, Helena C Loureiro, Rosalina Barroso

**Affiliations:** 1 Pediatric Service, Child and Youth Department, Hospital Professor Doutor Fernando Fonseca, Amadora, PRT; 2 Neonatology Service, Child and Youth Department, Hospital Professor Doutor Fernando Fonseca, Amadora, PRT; 3 Gynecology and Obstetrics Service, Women’s Department, Hospital Professor Doutor Fernando Fonseca, Amadora, PRT

**Keywords:** infant sleep, quality improvement projects, sleep habits, sleep position, s: sudden infant death syndrome

## Abstract

Introduction

Promoting healthy sleep practices from birth has a positive effect on infants and their families. Our goal was to implement measures to promote safe and healthy sleep practices for infants and to evaluate their impact.

Methods

A quality improvement project was developed in the maternity ward of a level II hospital in Portugal. The study comprised three phases: evaluation of the information provided to parents and infants’ sleep habits by a telephone survey between two and three months of age; intervention through the application of measures, which included informative leaflets, posters, and training sessions for medical and nursing staff; and evaluation of the implemented measures after one year, applying the same survey.

Results

We recorded an increase of 13% in parents informed about safe sleep surfaces (p=0.001) and position (p<0.001). Additionally, 19.3% more infants were sleeping in the supine position (p<0.001). Only 74.7% were sleeping in a crib, and bed sharing was still a common practice (20.9%). Regarding sleep habits, we recorded a 7.6% increase in infants sleeping in the dark at night (p=0.216), and a 17.2% increase in the use of the night light (p=0.003) for diaper changes/feeding.

Discussion

Our project was effective in improving parents’ knowledge concerning safe sleep practices, increasing the proportion of infants sleeping in the supine position, and reducing light exposure at night. However, we had no effect on bed sharing, which remains frequent, related to our population's cultural background.

## Introduction

Sleep is a necessary and essential function for life and one of the human resources for homeostasis [1-3]. The duration, quality, and structure of sleep evolve throughout the human lifespan, with particularly significant changes occurring during the first five years of life. Newborns spend up to 80% of the day asleep, while most infants and preschool-aged children sleep for up to 50% of the day [3]. Inadequate sleep, particularly at early ages, has adverse effects on overall health, endocrinological and metabolic functions, and also neurocognitive and emotional development [1,2,4-6]. There are cultural differences in the duration, patterns, and habits of sleep in infants and children [3,6,7]. Similarly, caregivers' perceptions and expectations regarding infants' sleep are influenced by cultural and family context, as well as by the media [8,9]. Promoting healthy sleep practices from birth has a positive effect on the quality of sleep for infants and their families [3,10,11]. These practices include establishing sleep routines; exposure to natural sunlight, with reduced lighting at night; bathing and/or massage at the end of the day; skin-to-skin contact; and reducing sound stimuli and activity in the hours before sleep [2,3].

Sudden unexpected infant death (SUID) is defined as the death of an infant less than one-year-old that occurs suddenly and unexpectedly. It includes sudden infant death syndrome (SIDS) when the cause remains unexplained after extensive investigation [12]. Since 1992, with the emergence of the first prevention campaigns, there has been a significant reduction in SUID and SIDS, with a trend towards stabilization from 2000 onwards. However, SIDS remains the leading cause of postnatal death (28 days per year) [12]. In 2019, the incidence of SUID in the United States of America (USA) was 90 per 100,000 live births [12]. In 2015, the incidence of SUID in Western Europe was 29.9 per 100,000 live births. In the European context, Portugal had one of the lowest rates, 16.4 per 100,000 live births [13]. Studies conducted in the USA showed ethnic and racial disparities. This is multifactorial and includes differences in the prevalence of infants sleeping in a supine position, the practice of bed-sharing being more common in African American families, and less access to economic, social, and educational resources [12].

Current recommendations for the prevention of SIDS include room-sharing with parents until six to 12 months, in their own crib; supine positioning without crib inclination; a firm mattress, well-fitted bedding without loose ends; avoiding overheating; and not placing stuffed animals, pillows, blankets, or other objects in the crib. Breastfeeding, especially exclusive breastfeeding, the use of a pacifier, and an up-to-date vaccination schedule are considered protective factors, while prematurity, low birth weight, pre-and/or postnatal exposure to tobacco, and caregivers under the influence of alcohol, drugs, or sedative medications are risk factors for SIDS [12].

The goal of this quality improvement project was to develop and implement measures to promote safe and healthy sleep practices in newborns or infants born in our hospital and to evaluate the impact of these measures. The hospital serves a population of 557,060 inhabitants, of which about 20% are under 20 years of age and 12% are foreign residents with legalized status [14]. During the years the study took place, 2021 and 2022, there were 2,148 and 2,647 births, respectively. Since 2010, the hospital has held certification as a Baby-Friendly Hospital. The maternity ward can accommodate up to 30 mother-infant dyads in a rooming-in arrangement.

## Materials and methods

This was a quality improvement, prospective, longitudinal, and interventional study developed by the maternity ward of Hospital Professor Doutor Fernando Fonseca, Amadora, a public level II hospital in Portugal. The study comprised three phases over a period of two years (2021-2022). In phase 1, we evaluated the information provided to parents during hospitalization and the sleep habits of infants born in January 2021, with a telephone survey to parents, applied when the child was two to three months old. The questions were designed by our team, based on variables identified through a literature review and clinical experience. It assessed demographic data, pregnancy and delivery data, feeding, knowledge concerning SIDS, measures to prevent SIDS, and infants’ sleep habits (Appendix 1).

**Figure 1:**
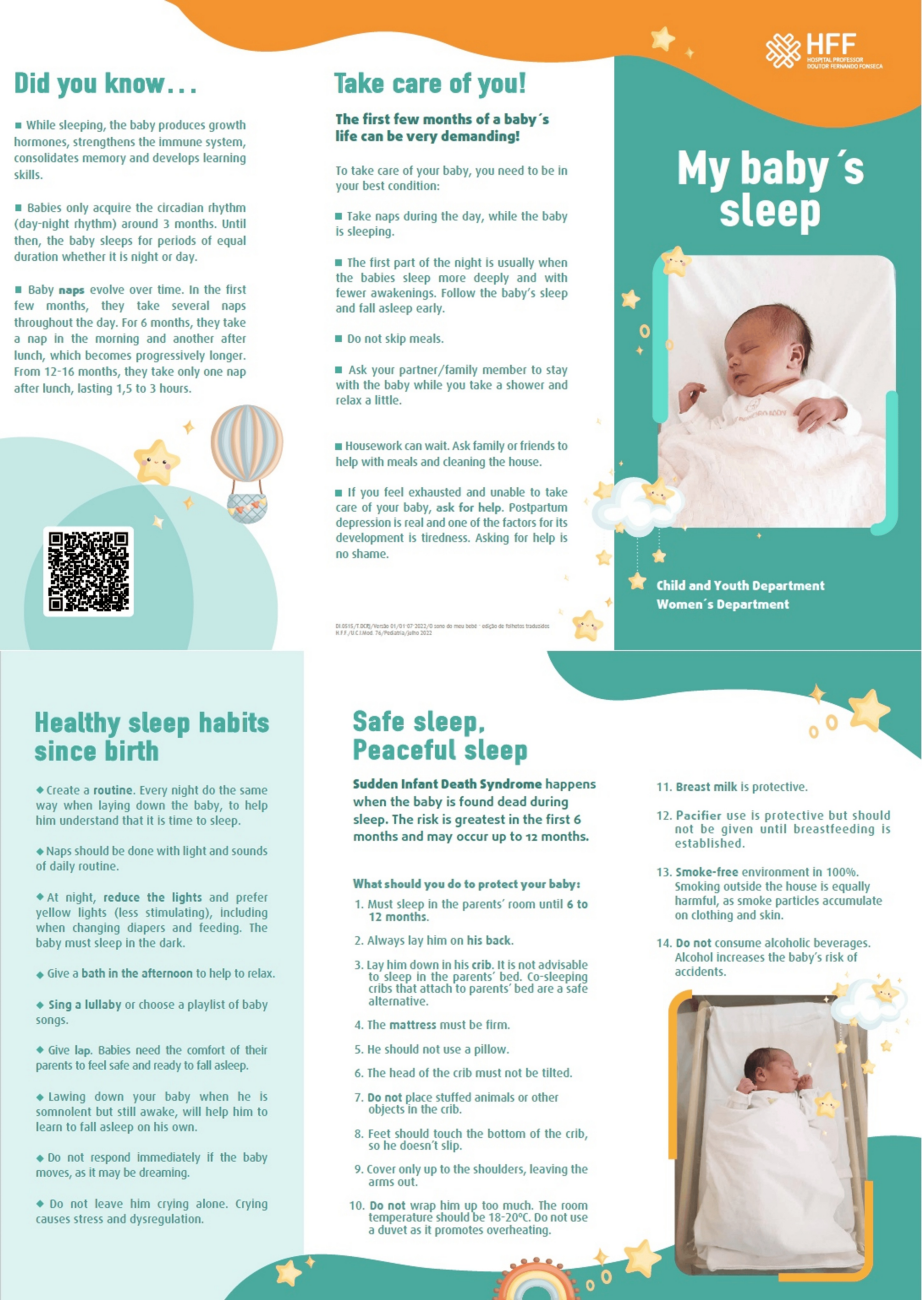
Informative leaflet (English version) Image credit: Hospital Professor Doutor Fernando Fonseca Team The parents of newborns photographed for posters and leaflets provided written informed consent.

**Figure 2:**
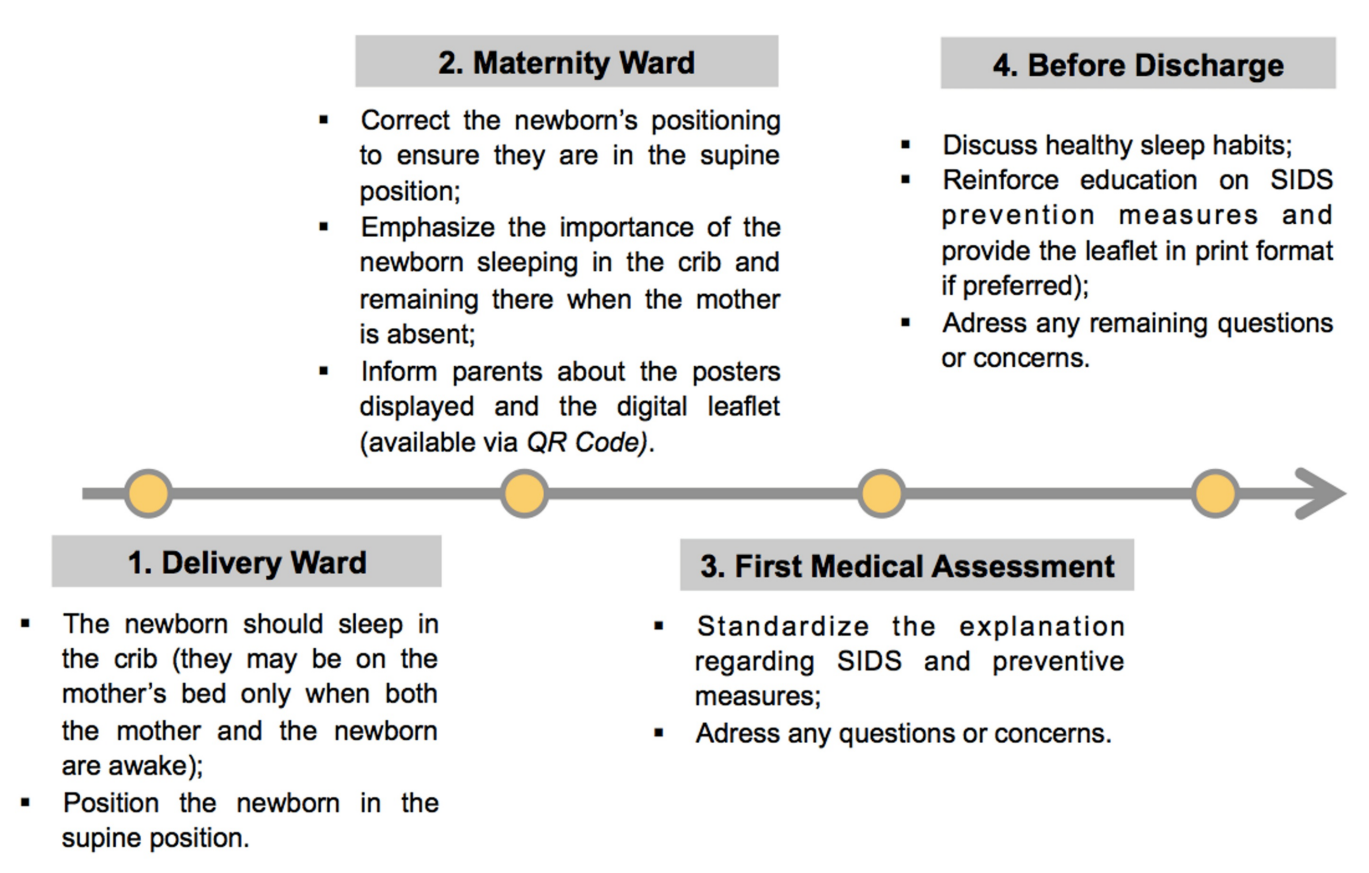
Intervention flowchart

In phase 2 (intervention), based on the analysis of the first surveys, measures were developed and implemented to improve the knowledge of parents about SIDS and to promote safe and healthy sleep practices. Informative leaflets (in Portuguese and English) and posters (in Portuguese) were created by our team and displayed in the maternity ward, obstetrics emergency, and consultation waiting rooms (Figure 1). Moreover, one training session was held for the neonatology medical staff and two for obstetrics nurse staff. These training sessions aimed to review current knowledge on healthy sleep habits in infants and on measures to prevent SIDS, standardize sleep practices in the maternity ward, and unify the information provided to parents. In this regard, we created an intervention flowchart with various stages, from the delivery ward to discharge from the maternity ward, to ensure that the medical and nursing teams could act in an integrated and standardized manner (Figure 2).

In phase 3, we evaluated the impact of these measures one year after being implemented. The same telephone survey was applied to parents of infants born in July 2022, at age two to three months old, similarly to phase 1. In both phases 1 and 3, the inclusion criteria were: newborns born in our hospital during the specified periods (January 2021 for phase 1 and July 2022 for phase 3); newborns rooming in with their mothers in the maternity ward; parents who speak Portuguese or English; parents who answered the telephone (three attempts on different days); and parents who provided verbal informed consent. The exclusion criteria included newborns born outside the specified periods; newborns admitted to the neonatal intensive care unit; language barriers (inability of the parents to communicate in English or Portuguese); parents who did not answer the telephone (three attempts on different days) or whose number listed in the records was invalid; and parents who did not provide informed consent for the survey. 

Lastly, we compared the data of the surveys applied in phases 1 and 3. The statistical analysis was performed using IBM SPSS Statistics for Windows, Version 28 (Released 2021; IBM Corp., Armonk, New York, United States). For continuous variables, the independent t-test was applied, while the Pearson chi-square test was employed for categorical variables. A significance level of <0.05 was set for all analysis.

The study was reviewed and approved by the hospital’s Ethics Committee (registration ID number 001/2022). The study has been granted an exemption from requiring written informed consent by the participants in the telephone surveys. The participants in the telephone surveys provided verbal informed consent. Written informed consent was provided by the parents of newborns photographed for posters and leaflets. All of the information was anonymous and confidential.

## Results

**Figure 3:**
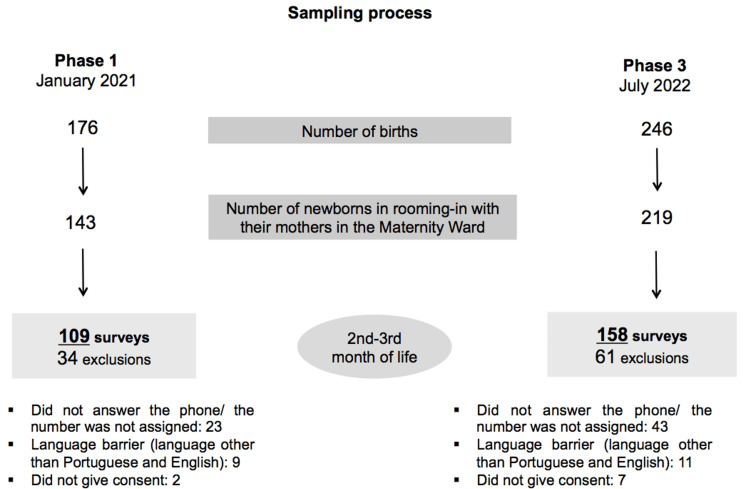
Sampling process

We conducted 109 surveys in phase 1 and 158 surveys in phase 3. The sampling process is described in Figure 3. The median age of the mothers was 31 years in both phases (Table 1). In phase 1, 59.6% of the mothers were Portuguese, and 40.4% were immigrants. In phase 3, we recorded a considerably higher number of immigrant mothers (70.3%), with only 29.7% being Portuguese. The most frequent countries of origin were Cape Verde, Guinea-Bissau, and Brazil. The median length of residence in Portugal was seven years in phase 1 and five years in phase 3. Regarding family structure, most were nuclear families (68.8% in phase 1, 80.4% in phase 3). The median number of household members was 4 in both phases.

**Table 1:** Demographic data

	Phase 1 (n=109)	Phase 3 (n=158)
Infant’s sex		
Male	52.3% (57)	48.7% (77)
Female	47.7% (52)	51.3% (81)
Mother’s age – median (minimum-maximum)	31 years (18-47 years)	31 years (18-46 years)
Mother’s nationality		
Portugal	59.6% (65)	29.7% (46)
Cape Verde	7.3% (8)	27.7% (43)
Guinea-Bissau	10.1% (11)	10.3% (16)
Angola	4.6% (5)	8.4% (13)
Sao Tome and Principe	4.6% (5)	4.5% (7)
Brazil	10.1% (11)	11.6% (18)
Others	3.6% (4)	7.8% (15)
Time living in Portugal – median (minimum-maximum)	7 years (1-31 years)	5 years (0-30 years)
Family structure		
Nuclear	68.8% (75)	80.4% (127)
Single parent	22.9% (25)	12.0% (19)
Extended	8.3% (9)	6.3% (10)
Reconstructed	0%	1.3% (2)
Number of household members – median (minimum-maximum)	4 members (2-10 members)	4 members (2-9 members)

Data related to pregnancy, delivery, and feeding are described in Table 2. Most pregnancies were single (96%) and full-term (92.7%). About 50% of deliveries were vaginal. At discharge, around 80% of newborns were exclusively breastfed. At the time of the survey, at two to three months of age, this percentage dropped to 56.0% in phase 1 and in lesser extent (65.8%) in phase 3.

**Table 2:** Pregnancy, delivery, and feeding data

		Phase 1 (n=109)	Phase 3 (n=158)
Type of pregnancy	Single	96.3% (105)	96.2% (152)
	Twin	3.7% (4)	3.8% (6)
Gestational age	Full-term	92.7% (101)	92.4% (146)
	Pre-term	7.3% (8)	7.6% (12)
Type of delivery	Vaginal	50.5% (55)	53.2% (84)
	Assisted vaginal (vacuum/ forceps)	17.4% (19)	13.9% (22)
	C-section	32.1% (35)	32.9% (52)
Skin-to-skin contact in the first hour of life	Yes	93.6% (102)	88.0% (139)
	No	6.4% (7)	12.0% (19)
Breastfeeding in the first hour of life	Yes	90.8% (99)	88.6% (140)
	No	9.2% (10)	11.4% (18)
Feeding on discharge	Breastfeeding	80.7% (88)	82.3% (130)
	Formula	3.7% (4)	3.8% (6)
	Combination	15.6% (17)	13.9% (22)
Feeding at the time of the survey	Breastfeeding	56.0% (61)	65.8% (104)
	Formula	19.3% (21)	13.9% (22)
	Combination	24.8% (27)	20.3% (32)

In phase 1, 80.7% of parents reported that during hospitalization they were informed about safe sleep surfaces, and 82.6% about safe sleep positions (Table 3). At the moment of the survey, 77.1% of infants were sleeping in a crib and 17.4% were bed sharing; 72.5% slept in the supine position; 58.7% of parents were familiar with the concept of SIDS, but only 25.7% knew up to what age it is most frequent; 57.8% of infants used a pacifier during sleep; 32.1% were exposed to tobacco smoke. In phase 3, we recorded an increase of around 13% in parents informed about safe sleep surface (p=0.001) and position (p<0.001). Additionally, 19.3% more infants were sleeping in the supine position (p<0.001). Nonetheless, only 74.7% were sleeping in a crib, and bed sharing was still a common practice (20.9%). Regarding knowledge of SIDS, we recorded an 11.6% increase in parents who were familiar with the concept (p=0.051), but similar rates related to the age up to which it is more frequent. We observed a 10.4% reduction in pacifier use (p=0.097) compared to phase 1 and a 12.5% reduction in tobacco exposure (p=0.020). Notably, in both phases, all infants were in room-sharing.

**Table 3:** Sudden infant death syndrome (SIDS) prevention

		Phase 1 (n=109)	Phase 3 (n=158)	Statistical analysis
During hospital stay, were you informed about safe sleep surface?	Yes	80.7% (88)	94.3% (149)	X^2^_(1)_=11.909; p=0.001
	No	19.3% (21)	5.7% (9)	
During hospital stay, were you informed about safe sleep position?	Yes	82.6% (90)	95.6% (151)	X^2^_(1)_=12.404; p<0.001
	No	17.4% (19)	4.4% (7)	
Do you know what is SIDS?	Yes	58.7% (64)	70.3% (111)	X^2^_(1)_=3.802; p=0.051
	No	41.3% (45)	29.7% (47)	
Do you know up to what age SIDS is most common?	Yes	25.7% (28)	25.9% (41)	X^2^_(1)_=0.002; p=0.962
	No	74.3% (81)	74.1% (117)	
Does the baby sleep in parents’ room?	Yes	100% (109)	100% (158)	-
	No	0% (0)	0% (0)	
In what position does the baby sleep most of the time?	Supine	72.5% (79)	91.8% (145)	X^2^_(1)_=17.773; p<0.001
	Prone	7.3% (8)	2.5% (4)	
	Lateral	20.2% (22)	5.7% (9)	
Where does the baby sleep most of the time?	Crib	77.1% (84)	74.6% (118)	X^2^_(1)_=0.198; p=0.656
	Bed sharing	17.4% (19)	20.9% (33)	
	Carrycot	5.5% (6)	4.5% (7)	
Does the baby use a pacifier to sleep?	Yes	57.8% (63)	47.4% (75)	X^2^_(1)_=2.756; p=0.097
	No	42.2% (46)	52.5% (83)	
Is the baby exposed to tobacco?	Yes	32.1% (35)	19.6% (31)	X^2^_(1)_=5.407; p=0.020
	No	67.9% (74)	80.4% (127)	

There was no statistically significant association between the nationality of the mother (Portuguese or immigrant) and parents being informed about safe sleep surfaces (p=0.526) or positions (p=0.419). Similarly, we found no association between the nationality of the mother and the surface (p=0.210) and position (p=0.907) the infant usually sleeps. Conversely, being an immigrant mother was associated with not knowing what SIDS is (p<0.001) and up to what age it is most frequent (p<0.001). We also identified a statistical association between being a Portuguese mother and the use of a pacifier (p<0.001) and exposure to tobacco smoke (p<0.001).

Regarding sleep habits (Table 4) and related routines, in both phases, most families preferred the evening for bath time (71.6% and 65.2%, respectively). Most infants slept during the day with natural light (84.4% and 86.0%, respectively). At night, in phase 1, only 37.6% of infants slept without light, in contrast with 6.4% and 4.6% that slept with screen light and lamp light, respectively. In phase 3, we recorded an 8.0% increase in infants sleeping without light (p=0.216), due to a decrease in screen light (4.4%) and lamp light (0.6%). For diaper changes or feeding during the night, in phase 1, a night light was used in 56.9%, screen light in 15.6%, and lamp light in 27.5%; in phase 3, we observed a 17.2% increase in the use of the night light (p=0.003), in detriment to screen and lamp light. Finally, regarding the duration and continuity of nighttime sleep, the median number of consecutive sleep hours was four and five hours, respectively. The median number of nighttime awakenings was one awakening per night in both phases.

**Table 4:** Sleep habits

		Phase 1 (n=109)	Phase 3 (n=158)	Statistical analysis
What time does the baby usually take a bath?	Evening	71.6% (78)	65.2% (103)	X^2^_(1)_=1.199; p=0.274
	Morning	11.9% (13)	13.3% (21)	
	Afternoon	2.8% (3)	6.3% (10)	
	Twice a day	3.7% (4)	8.9% (14)	
	Variable	10.1% (11)	6.3% (10)	
Does the baby take naps in daylight?	Yes	84.4% (92)	86.0% (136)	X^2^_(1)_=1.450; p=0.704
	No	15.6% (17)	13.9% (22)	
Does the baby sleep with a light at night?	Night light	51.4% (56)	49.4% (78)	X^2^_(1)_=1.528; p=0.216
	Screen light	6.4% (7)	4.4% (7)	
	Lamp	4.6% (5)	0.6% (1)	
	No	37.6% (41)	45.6% (72)	
What light do you use to feed or change the baby’s diaper at night?	Night light	56.9% (62)	74.1% (117)	X^2^_(1)_=8.606; p=0.003
	Screen light	15.6% (17)	19% (30)	
	Lamp	27.5% (30)	6.9% (11)	
How many hours does the baby sleep at a time? – median (minimum-maximum)		4 hours (2-12 hours)	5 hours (1-9 hours)	t_(264)=0.131;_ p=0.896
How many times does the baby wake up at night? – median (minimum-maximum)		1 time (0-4 times)	1 time (0-5 times)	t_(265)=0.260;_ p=0.795

## Discussion

The results before quality measures implementation (phase 1) demonstrated unsafe sleep practices and gaps in parents' knowledge about SIDS. Regarding the role of healthcare professionals, the results show that information about safe sleep practices was regularly provided to parents in the vast majority of cases. After the implementation of the quality measures (phase 3), we noted a significant increase in the rate of parents informed about safe sleep practices and infants sleeping in the supine position. However, the measures did not significantly impact bed-sharing practices. Notably, we did not identify significant differences between the immigrant and Portuguese populations in terms of positioning babies or bed sharing. We acknowledge that cultural factors associated with bed-sharing are very strong in our population (both Portuguese and immigrant), making this aspect difficult to change through the measures applied in this study.

Regarding knowledge of SIDS, we also observed an increase (though not statistically significant) in the rate of parents aware of this concept, but not concerning the age up to which it is most frequent. In the immigrant population, literacy regarding SIDS was lower. In phase 3, the rate of immigrants was higher than in phase 1, which we believe may have contributed to a lesser effect of the implemented measures on SIDS acquaintance. Cultural differences were also noticeable in the use of pacifiers and exposure to tobacco smoke, which were both more frequent in the Portuguese population. Therefore, we believe that the reduction in these variables from phase 1 to 3 is not related to our intervention but rather to the higher prevalence of the immigrant population in phase 3.

Regarding light exposure, the measures were effective in increasing the use of night lights for diaper changes or feedings during the night, instead of using more intense lights. Although not statistically significant, we also noted an increase in the rate of infants sleeping at night without light. Studies show that cyclical light exposure according to the day-night pattern is important from early ages to establishing a sleep routine [2,3]. Infants who have a cycled light pattern tend to sleep longer during the night period [2]. Regarding the duration and continuity of nighttime sleep, our results align with the physiology of sleep in infants, which involves frequent nighttime awakenings for feeding, given the high energetic needs [8].

We found several quality improvement studies in the literature targeting safe and healthy sleep practices in infants. In the study published by Shaikh SK et al., the implemented quality measures were based on educating the nursing staff and parents, as well as providing sleep sacks for the infants. After a year, the percentage of infants with 'perfect sleep' increased from a baseline of 41.9% to 67.3%. Improvements were sustained over 12 months later, suggesting that quality improvement interventions can have long-lasting results [15]. Another study by Kellams et al., which involved eight maternity hospitals in the United States, used a set of materials that included, among others, posters and pocket-size cards for parents. It also showed positive results, with an increase of 24-57% from baseline in the percentage of mothers who reported receiving information about safe sleep, an increase of 24% in infants sleeping in the supine position (93%), and of 33% in those sleeping in a safe sleep environment (88%). Changes were also sustained for up to 12 months [16]. The study by Mason B. et al. also used an intervention bundle with educational tools for parents. In the initial phase, only 25% of infants had safe sleep practices. After the intervention, the percentage increased to 58%, and 95% of parents planned to use the supine position at home [17]. A study conducted in Portugal by Martins et al. compared the sleep habits of infants whose mothers received information in the maternity ward about sleep hygiene versus mothers who did not receive that information. Maternal education was positively associated with healthy and autonomous sleep habits in infants up to six months of age, namely sleeping in their own crib and falling asleep on their own. The authors propose that sleep education should start as early as possible, ideally during pregnancy and early postpartum [11]. Our study, similar to those mentioned above, reinforces the effectiveness of quality improvement programs in promoting safe and healthy sleep habits in infants, especially if starting with early instruction of the families. 

We identified some limitations in the study. Our leaflets were available in Portuguese and English, and the posters were only in Portuguese. The population served by our hospital is characterized by a high rate of immigrants, and although most come from Portuguese-speaking countries, many are from other countries. The language barrier may have limited our intervention, not only in the leaflets/posters but also in the oral transmission of information to parents. It is noteworthy that the telephone surveys were conducted in Portuguese or English, thus excluding participants not fluent in these languages, which reduces bias in the study results. In the near future, we consider it a priority to translate the materials into the most prevalent native languages in our population, namely French and Creole languages. The samples from phases 1 and 3 have some important differences, not only in number (more births and consequently more surveys conducted in phase 3) but also in a higher proportion of immigrant mothers in phase 3. Cultural factors associated with sleep, as well as the language barrier, may therefore have had a greater impact in phase 3. The surveys were conducted by telephone by only one of the study authors, which reduced subjectivity and variability in the survey application among the participants. However, we do not exclude the possibility that response induction may have occurred, especially on questions perceived as sensitive, such as smoking, which may therefore be underestimated. Finally, in the survey variables, we did not include the educational level of mothers, a potentially important factor for understanding sleep-related literacy.

It is important to highlight that the surveys also provided an opportunity to clarify doubts and to educate the families. All parents were informed about safe sleep surfaces and positions, recommendations for the prevention of SIDS, and the importance of proper cyclical light exposure for the establishment of a sleep routine.

The training sessions conducted for the medical and nursing staff aimed to standardize the sleep practices in the maternity ward, as well as the information transmission to parents. However, team turnover, especially in the nursing team, is a factor associated with information transmission failures, which may have been a limitation in the implementation of this project, requiring ongoing training.

## Conclusions

In conclusion, the quality measures implemented were effective in improving parents’ knowledge concerning safe sleep practices, in increasing the proportion of infants sleeping in the supine position, and in reducing light exposure at night. Nonetheless, the measures had no statistical effect on bed sharing, which remains very frequent and worrisome. Although it is highly not recommended, we believe that cultural factors associated with bed-sharing are still very strong in our population, making this aspect difficult to change through the measures applied in the study. Establishing healthy sleep practices from birth has a positive impact throughout life. With this project, our hospital prioritizes sleep as one of its healthcare quality and patient education goals.

## Appendices

Appendix 1

Telephone Survey Consent Form

With this telephone survey, we are inviting you to participate in a quality improvement project aimed at implementing measures to promote safe and healthy sleep practices for infants and evaluating the impact of these measures.

Your participation is entirely voluntary. The survey will take approximately 10 minutes to complete. We will ask you questions about demographic data, pregnancy and delivery, feeding, knowledge concerning sudden infant death syndrome (SIDS), measures to prevent SIDS, and infants' sleep habits. You may skip any question you do not wish to answer, and you can stop the survey at any time without any consequences.

Your responses will remain confidential and will be used solely for research purposes. No personally identifiable information will be linked to your responses.

There are no known risks associated with participating in this survey. While there are no direct benefits to you, your feedback will contribute to improving the care provided in the maternity ward of our hospital, specifically regarding infant sleep.

Your participation is entirely voluntary. You are free to decline or withdraw at any point during the survey without any penalty or loss of benefits.

By verbally consenting to participate in this survey, you are confirming that you have understood the purpose of the survey and what participation involves, that your participation is voluntary, and that you may withdraw at any time or choose not to answer specific questions.

Telephone Survey

Date of birth: ___ /___ / ________ 

Date of telephone survey: ___ /___ / _________

1. Demographic data

1.1. Infant’s sex: 

□   Male

□   Female

1.2. Mother’s age:  _________

1.3. Mother’s nationality: ___________

1.4. Time living in Portugal (in case of immigrant mother): ___________

1.5. Family structure:

□   Nuclear

□   Extended

□   Single parent

□   Reconstructed

1.6. Number of household members: ___________

2. Pregnancy, delivery, and feeding 

2.1. Type of pregnancy:

□   Single

□   Twin

2.2. Gestacional age: ________

2.3. Type of delivery: 

□   Vaginal

□   Assisted vaginal (vaccum/ forceps)

□   C-section

2.4. Did the baby have skin-to-skin contact in the first hour of life?

□   Yes

□   No

2.5. Was the baby breastfed within the first hour of life?

□   Yes

□   No

2.6. What was the type of feeding at the time of discharge?

□   Breastfeeding

□   Formula

□   Combination

2.7. What is the current type of feeding?

□   Breastfeeding

□   Formula

□   Combination

3. Sudden infant death syndrome (SIDS) prevention

3.1. During hospital stay, were you infomed about safe sleep surface?

□   Yes

□   No

3.2. During hospital stay, were you infomed about safe sleep position?

□   Yes

□   No

3.3. Do you know what is SIDS?

□   Yes

□   No

3.4. Do you know up to what age SIDS is most common?

□   Yes

□   No

3.5. Does the baby sleep in parent’s room?

□   Yes

□   No

3.6. In what position does the baby sleep most of the time?

□   Supine

□   Prone

□   Lateral

3.7. Where does the baby sleep most of the time?

□   Crib

□   Bed sharing

□   Carrycot

3.8. Does the baby use a pacifier to sleep?

□   Yes

□   No

3.9. Is the baby exposed to tobacco?

□   Yes

□   No

4. Sleep habits

4.1. What time does the baby usually take a bath?

□   Evening

□   Morning

□   Afternoon

□   Twice a day

□   Variable

4.2. Does the baby take naps in daylight?

□   Yes

□   No

4.3. Does the baby sleep with a light at night?

□   Night light

□   Screen light

□   Lamp

□   No

4.4. What light do you use to feed the baby or change the baby’s diaper at night?

□   Night light

□   Screen light

□   Lamp

4.5. How many hours does the baby sleep at a time? _________

4.6. How many times does the baby wake up at night? ________
